# Activation Enhancement and Grain Size Improvement for Poly-Si Channel Vertical Transistor by Laser Thermal Annealing in 3D NAND Flash

**DOI:** 10.3390/mi14010230

**Published:** 2023-01-16

**Authors:** Tao Yang, Zhiliang Xia, Dongyu Fan, Dongxue Zhao, Wei Xie, Yuancheng Yang, Lei Liu, Wenxi Zhou, Zongliang Huo

**Affiliations:** 1 Institute of Microelectronics of the Chinese Academy of Sciences, Beijing 100029, China; 2 University of Chinese Academy of Sciences, Beijing 100049, China; 3 Yangtze Memory Technologies Co., Ltd., Wuhan 430205, China

**Keywords:** 3D NAND Flash, vertical channel transistor, laser thermal annealing, dopant activation, Poly-Si, grain size

## Abstract

The bit density is generally increased by stacking more layers in 3D NAND Flash. Lowering dopant activation of select transistors results from complex integrated processes. To improve channel dopant activation, the test structure of vertical channel transistors was used to investigate the influence of laser thermal annealing on dopant activation. The activation of channel doping by different thermal annealing methods was compared. The laser thermal annealing enhanced the channel activation rate by at least 23% more than limited temperature rapid thermal annealing. We then comprehensively explore the laser thermal annealing energy density on the influence of Poly-Si grain size and device performance. A clear correlation between grain size mean and grain size sigma, large grain size mean and sigma with large laser thermal annealing energy density. Large laser thermal annealing energy density leads to tightening threshold voltage and subthreshold swing distribution since Poly-Si grain size regrows for better grain size distribution with local grains optimization. As an enabler for next-generation technologies, laser thermal annealing will be highly applied in 3D NAND Flash for better device performance with stacking more layers, and opening new opportunities of novel 3D architectures in the semiconductor industry.

## 1. Introduction

With the continuous development of smartphones, 5G, and data centers, the market demand for higher bit density has grown rapidly. The bit density is generally increased by stacking more layers in 3D NAND Flash [1,2,3,4,5,6,7,8]. However, stacking more layers makes integrated processes more complex, leading to worse wafer stress [9,10,11] and leakage caused by fluorine attack [12]. Meanwhile, the doping profile of vertical select transistors needs to be well controlled for better performance. This limits the high-temperature process for the vertical select transistor channel dopant activation. Therefore, the vertical select transistor must be forced to accept the low temperature to activate the channel dopant. To transition to the higher dopant activation process, laser thermal annealing (LTA) can achieve and meet the requirements of a low thermal budget. The LTA is well applied in power devices extending the Si-based devices with μm-scale deep activation [13,14,15], CMOS logic, and 3D sequential integration for active area formation and source/drain activation [16,17,18]. However, LTA has been less studied for memory applications compared with these fields. In the DRAM field, polysilicon contact plug annealing [19] is considered to remove voids with LTA. While in 3D NAND Flash, Lisoni et al. proposed the use of LTA to crystallize amorphous silicon channel in vertical channel transistors [20], thus optimizing the grain size. Son et al. used numerical simulation to study the laser conditions with multipath and beam overlap to improve temperature uniformity within the annealed area [21]. However, channel dopant activation in vertical transistors has not been studied. Therefore, it is essential to study the dopant activation and Poly-Si channel grains of vertical channel transistors for 3D NAND Flash application.

In this work, we demonstrated the dopant activation and the engineering of Poly-Si channel grains in vertical channel transistor devices by laser thermal annealing in 3D NAND Flash. The activation of channel dopant by different thermal annealing methods was compared, and the results show that the LTA enhanced the channel activation rate by at least 23% more than RTA. We then comprehensively explored the LTA energy density’s influence on Poly-Si grain size and device performance. A clear correlation was discovered between grain size mean and grain size sigma, large grain size mean and sigma with large LTA energy density. Large LTA energy density leads to tightening threshold voltage and subthreshold swing distribution, which is by Poly-Si grain size regrowth for better grain size distribution with local grains optimization by larger energy density LTA.

## 2. Experiments

**Figure 1:**
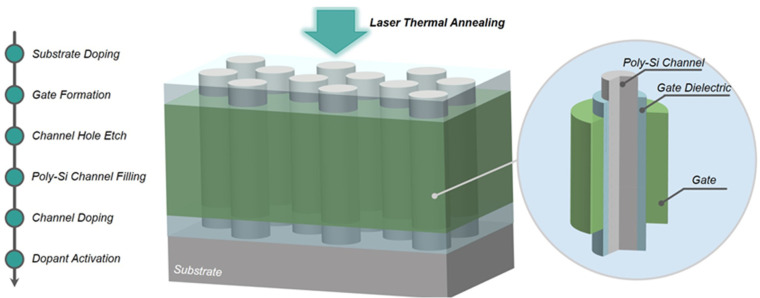
The main fabrication steps of the vertical channel transistor test structure. The schematic diagram of laser thermal annealing vertical channel transistors array. The inset shows the detailed structure of the vertical channel transistor.

Figure 1 outlines this work’s main fabrication steps of the vertical channel transistor test structure. The inset shows the detailed structure of the vertical channel transistor. The Poly-Si channel is filled, the ion implantation of boron is used for channel doping, and then drain doping formation occurs and low temperature rapid thermal annealing (RTA) within seconds or through nanosecond laser thermal annealing, which was followed by activation and annealing. In order to determine the LTA energy density condition, the maximum energy density is determined by the temperature of the substrate, which will not cause a fluorine attack problem in 3D NAND Flash and does not lead to cracks and loss of continuity of the dielectric films. The minimum energy density is determined by the channel dopants that were activated for electrical requirements. Therefore, the LTA energy density range was simulated based on well calibrated COMSOL Multiphysics software. The Synopsys Sentaurus TCAD extracted the dopant activation rate based on the dopant’s secondary ion mass spectroscopy (SIMS) results in the Poly-Si channel. The Keysight B1500 semiconductor parameter analyzer was used for electrical measurements. The Poly-Si grain is characterized by Transmission Electron Microscope (TEM) and, with an nm-scale precision, Precession Electron Diffraction (PED) technology.

## 3. Results and Discussion

**Figure 2:**
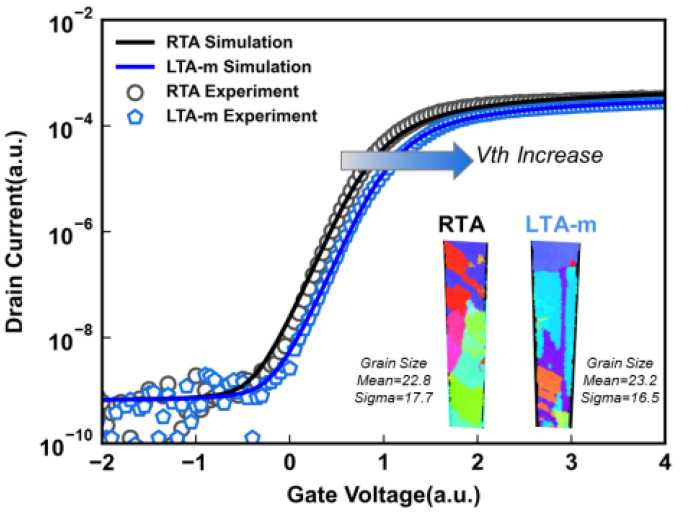
The Id-Vg characteristics of the RTA and LTA-m. The inset is the PED graphs of the cross-section view of the Poly-Si channel with RTA and LTA-m and the grain size mean value of RTA and LTA-m.

**Figure 3:**
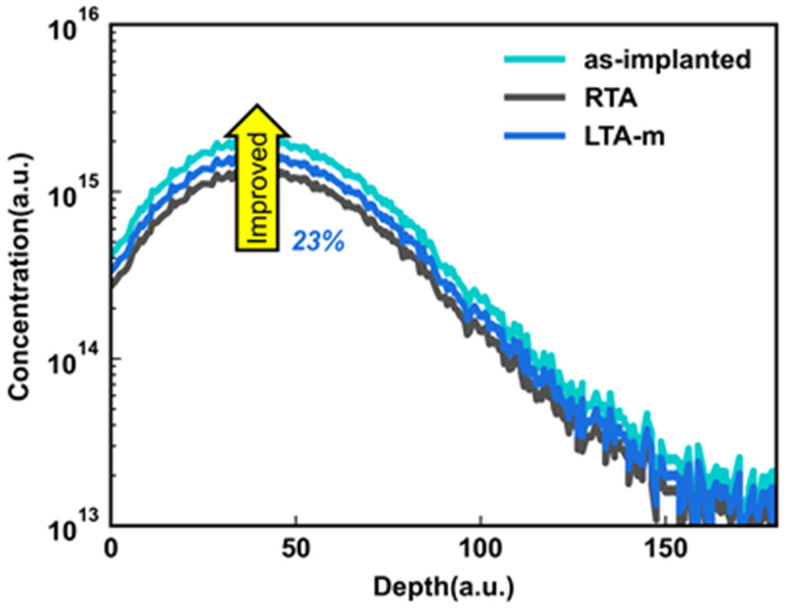
Measured boron concentration after ion implantation as implanted SIMS. Simulated the boron concentration of subsequent activation with RTA and LTA-m by Sentaurus TCAD.

Figure 2 shows the Id-Vg characteristics of the RTA and LTA m J/cm^2^(LTA-m). LTA-m shows a higher threshold voltage (Vth). To determine the cause, the PED is used to quantify the grain size of analyzed samples, and the LTA-m obtains a larger grain size than RTA. Then the grain size distribution was applied to the Sentaurus TCAD simulation based on [22], and the dopant activation rate of fitted Id-Vg curves was extracted. The simulation shown in Figure 3 shows that the boron activation rate of LTA-m is at least 23% higher than the RTA method. The enlarged grain size with fewer grain boundaries leads to fewer traps. This indicates that the LTA can enhance dopant activation. Meanwhile, the Poly-Si recrystallization can be enhanced for larger grain sizes.

**Figure 4:**
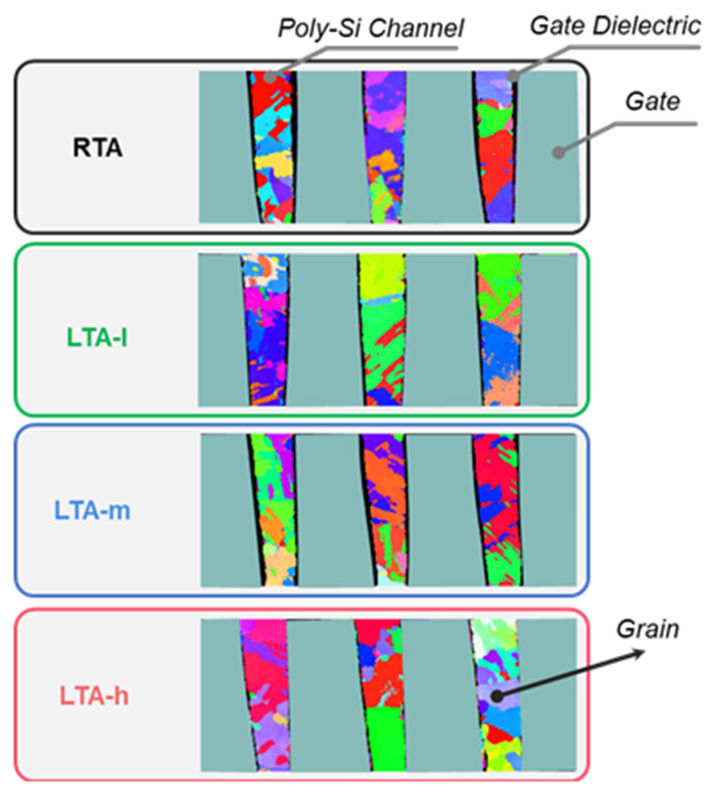
The PED graphs of the cross-section view of Poly-Si with different thermal conditions, RTA, LTA-l, LTA-m, and LTA-h.

**Figure 5:**
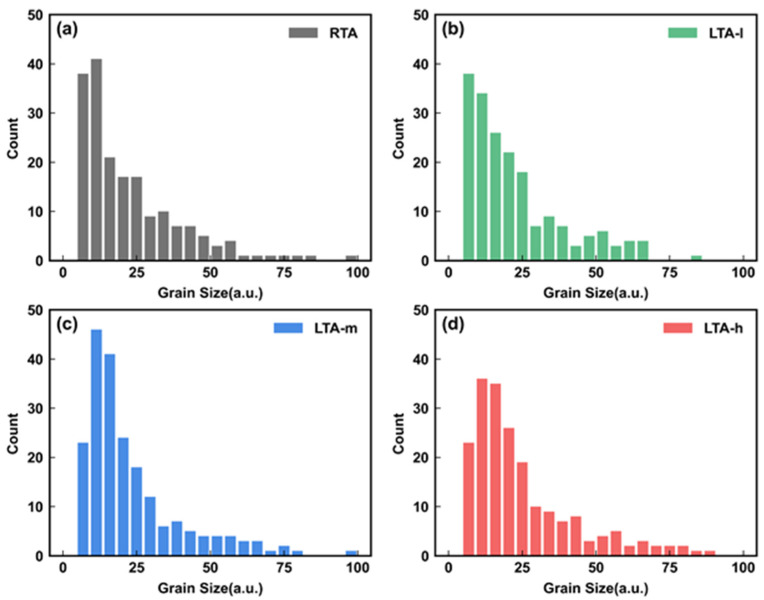
The statistics of grain size distribution with different thermal conditions, (**a**) RTA, (**b**) LTA-l, (**c**) LTA-m, and (**d**) LTA-h.

In order to investigate the influence of different LTA energy densities within the safety range on the Poly-Si channel, we chose three LTA energy density values of l J/cm^2^(LTA-l), m J/cm^2^(LTA-m) and h J/cm^2^(LTA-h), wherein, l < m < h. Figure 4 shows the PED graphs of the cross-section view of Poly-Si with different thermal conditions. In order to maintain the stability of data statistics, more than eight channel holes are required, and the grain size distribution is statistically stable to extract the grain size distribution. Then, the grain size distribution is shown in Figure 5. The LTA energy density increases, leading to larger grain size. It can be seen that a larger LTA energy density mainly leads to small grains regrowth. The larger the LTA energy density leads to small-sized grains. Meanwhile, the large grains of each LTA energy density on the right show almost no growth. The absorbed energy may be more helpful to the growth of small grains due to the short laser time, which means local grain size optimization.

**Figure 6:**
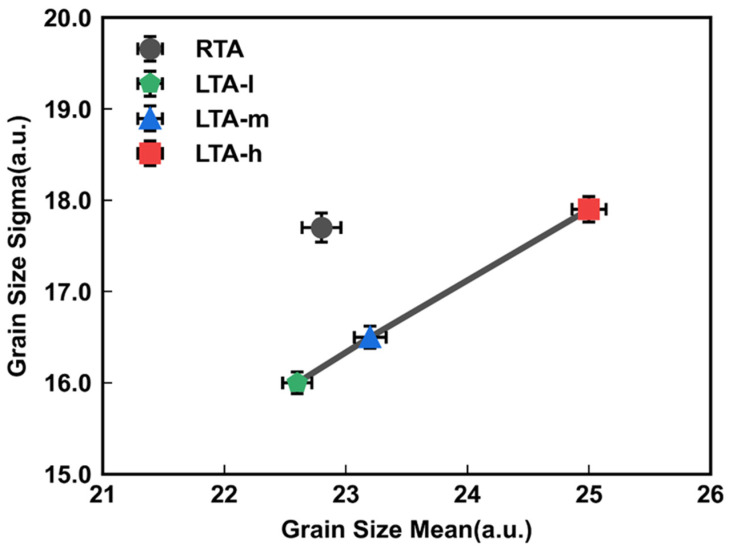
The correlation between grain size mean and grain size sigma of the Poly-Si channel with different thermal conditions, RTA, LTA-l, LTA-m, and LTA-h. The bars represent mean values, with the error bars showing standard deviation.

Figure 6 shows the correlation between grain size mean and grain size sigma. The larger grain size mean leads to the larger grain size sigma with the same thermal method. The RTA method has a larger grain size sigma under the same grain size mean. That is, different annealing methods lead to different recrystallization states of Poly-Si. The LTA method can improve the uniformity of Poly-Si grain size while increasing the grain size mean. The RTA method has worse grain size distribution than the LTA since the RTA forms more nucleating sites leading to more small grains, while LTA may form fewer nucleating sites. Meanwhile, the laser energy may be localized, so the energy provided is more helpful to the growth of small grains. Therefore, the LTA method is helpful for the growth of Poly-Si grains with better distribution.

**Figure 7:**
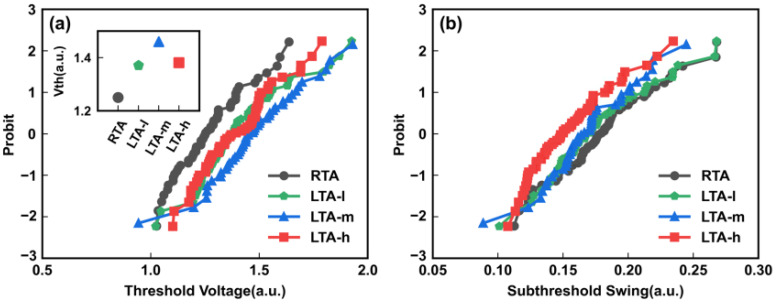
(**a**) Threshold voltage, the inset shows the correlation between LTA energy density and threshold voltage, (**b**) Subthreshold swing and Ion distributions of vertical channel transistors with different thermal conditions, RTA, LTA-l, LTA-m, and LTA-h.

Next, the influence of different LTA energy densities on the electrical characteristics of vertical channel transistors is studied. The electrical measurements come from more than 50 vertical transistors of each die. The statistical distribution of Vth and subthreshold swing(SS) are shown in Figure 7. A clear inverted U-shaped trend between LTA energy density and Vth, the largest LTA energy density LTA-h, shows a smaller Vth in the inset of Figure 7a. The Vth is affected by the activation rate of boron in the channel and traps in the Poly-Si channel. A larger activation rate leads to a larger Vth. Larger grain size with fewer traps leads to smaller Vth. After the energy density reaches LTA-h, the traps in the Poly-Si channel are significantly reduced in larger grain size, leading to a smaller Vth. Finally, the inverted U-shaped trend is formed, while in Figure 7b, the trend of SS is related to the grain size, and a clear improvement is made in SS with the larger LTA energy density. Consequently, LTA improves the grain size distribution in the boron-doped Poly-Si channel. The dose of channel doping with LTA could be further optimized to minimize the large dose impact. The vertical channel transistor could obtain better performance.

## 4. Conclusions

We have demonstrated the dopant activation and the engineering of Poly-Si channel grains in vertical channel transistor devices by laser thermal annealing in 3D NAND Flash. The LTA enhanced the channel activation rate by at least 23% more than RTA, leading to a tightening threshold voltage and subthreshold swing distribution, which is caused by Poly-Si grain size regrowth for better grain size distribution with local grains optimization. The laser thermal annealing process with energy density LTA-h can be an enabler for improving device performances. Laser thermal annealing will be highly applied in 3D NAND Flash for next-generation technologies with stacking more layers, opening new opportunities for novel 3D architectures in the semiconductor industry.

## Data Availability

The data presented in this study are available on request from the corresponding author. The data are not publicly available due to confidentiality request.
